# Rhamnolipid Biosurfactants as New Players in Animal and Plant Defense against Microbes

**DOI:** 10.3390/ijms11125095

**Published:** 2010-12-09

**Authors:** Parul Vatsa, Lisa Sanchez, Christophe Clement, Fabienne Baillieul, Stephan Dorey

**Affiliations:** Reims Champagne-Ardenne university, URVVC-SE-EA 2069, stress, defense and plant reproduction laboratory, BP 1039, F-51687 Reims cedex 2, France

**Keywords:** rhamnolipids, plant immunity, animal immunity, antimicrobial properties

## Abstract

Rhamnolipids are known as very efficient biosurfactant molecules. They are used in a wide range of industrial applications including food, cosmetics, pharmaceutical formulations and bioremediation of pollutants. The present review provides an overview of the effect of rhamnolipids in animal and plant defense responses. We describe the current knowledge on the stimulation of plant and animal immunity by these molecules, as well as on their direct antimicrobial properties. Given their ecological acceptance owing to their low toxicity and biodegradability, rhamnolipids have the potential to be useful molecules in medicine and to be part of alternative strategies in order to reduce or replace pesticides in agriculture.

## 1. Introduction

**Figure 1.**
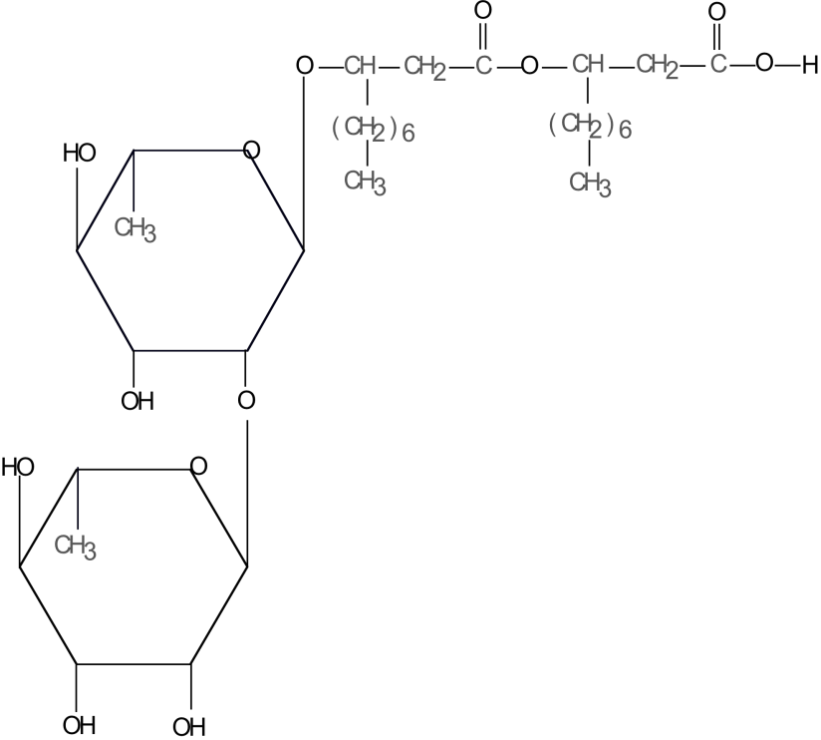
The major form of rhamnolipid produced by *Pseudomonas aeruginosa* (Rha-Rha-C_10_-C_10_).

Rhamnolipids (RLs) are glycolipid biosurfactants produced by various bacterial species including some *Pseudomonas* sp. and *Burkholderia* sp. [1]. The structure of RLs is highly diverse and those produced by *Pseudomonas aeruginosa* have been extensively studied. These RLs are amphiphilic molecules typically composed of 3-hydroxyfatty acids linked through a beta-glycosidic bond to mono- or di-rhamnoses (Figure 1) [2]. RLs have several potential functions in bacteria. They are involved in the uptake and biodegradation of poorly soluble substrates and are essential for surface motility and biofilm development [1]. From a biotechnological point of view, RLs are powerful biosurfactants with applications related to environmental concerns, such as bioremediation of hydrocarbon, organic pollutants and heavy-metal-contaminated sites. These topics have been extensively reviewed including some very recent articles [3–6]. RLs have also been used in the production of fine chemicals, surface coatings, as well as additives for food and cosmetics [7]. Finally, a new role for RLs as potential players in the combat of plants and animals against microbes has recently emerged. For years RLs have been extensively studied regarding their direct toxicity to microorganisms but recently they have also been reported to be involved in the stimulation of plant and animal defense responses. The present review provides an update of the current knowledge on the antimicrobial properties of RLs and also highlights the recent discoveries of the involvement of these molecules in the stimulation of immunity in plants and animals. The potential use of these molecules to fight against pathogenic microorganisms in medical and agricultural field will be discussed.

## 2. Rhamnolipids as Antimicrobial Agents

RLs have been shown to display antibacterial activities against plant and human pathogenic bacteria. RLs are known to be active against the Gram-negative bacteria *P. aeruginosa*, *Enterobacter aerogenes*, *Serratia marcescens* and *Klebsiella pneumonia*, as well as against Gram-positive *Micrococcus* sp., *Streptococcus* sp., *Staphylococcus* sp. and *Bacillus* sp [8–13] (Table 1). RLs have direct impact on bacterial cell surface structures. Al-Tahhan *et al*. [14] observed a loss of lipopolysaccharides (LPS) in *P. aeruginosa* strains treated with RLs at low concentrations and this resulted in increased cell surface hydrophobicity. Recently, Sotirova *et al*. [15] showed that RLs from *Pseudomonas* sp. PS-17 interact with *P. aeruginosa* causing a reduction in LPS content and changes in the outer membrane proteins of the bacteria. These changes had a direct impact on bacterial cell surface morphology. Sotirova *et al*. [15] concluded that RLs from *Pseudomonas* sp. PS-17 have a potential application in the field of biomedicine against pathogenic bacteria. Several studies described antifungal activity of RLs mainly against phytopathogens including *Botrytis* sp., *Rhizoctonia* sp., *Pythium* sp., *Phytophtora* sp. and *Plasmopara* sp. (Table 1) [16–22]. Additionally, RLs were also shown to be active against *Mucor miehei* and *Neurospora crassa* [12]. The main mode of action of RLs against zoospore-producing plant pathogens is the direct lysis of zoospores via the intercalation of RLs within plasma membranes of the zoospore which are not protected by a cell wall [16,21,23]. Recent studies also demonstrated an effect of RLs in the reduction of mycelia growth of *Pythium myriotylum* [18] and *Botrytis cinerea* [23]. These data suggest that RLs may also have an adverse effect on cell structures that are protected by a cell wall. Properties of RLs against the algae *Heterosigma akashiwo*, viruses, amoeba like *Dictyostelium discoideum* and mycoplasma have also been reported [24–29]. However, RLs’ applications have no significant effects on yeasts [10,12,17,28]. In addition to their *in vitro* antimicrobial activity, RLs have proven to be also efficient in *in vivo* plant systems. Treatments with RLs have been shown to protect pepper plants from *Phytophthora* blight disease and also prevent the development of *Colletotrichum orbiculare* infection on leaves of cucumber plants [17]. Yoo *et al*. [22] investigated RLs as alternative antifungal agents against typical plant pathogenic oomycetes, including *Phytophthora* sp. and *Pythium* sp. They showed that RLs significantly decrease the incidence of water-borne damping-off disease. Sharma *et al*. [19] obtained similar results in field trials on chili pepper and tomato. Using bacterial mutants, Perneel *et al*. [18] clearly showed that phenazine and RLs interact in the biological control of soil-borne diseases caused by *Pythium* spp. Recent studies also demonstrated that a combination mixture of SRE (Syringomycin E) and RLs is efficient against pathogenic and opportunistic fungi recovered from diseased grape [30,31].

## 3. Rhamnolipids in Plant and Animal Immunity

During the last decade, pattern recognition emerged as a fundamental process in the immune response of plants and animals. Perception by pattern recognition receptors (PRRs) of molecular signatures that identify whole classes of microbes but are absent from the host allows this nonself recognition [32,33]. Once recognized, these molecular signatures, conventionally named microbe-associated molecular patterns (MAMPs) [34], trigger complex signaling pathways leading to transcriptional activation of defense-related genes and accumulation of antimicrobial metabolites in plant cells [32]. In mammals, MAMP perception leads to the inflammatory response with the production of cytokines including interleukins and the tumor necrosis factor α (TNFα). Years ago, lipopeptides were shown to stimulate human innate immune responses through the PRR Toll-like receptor TLR2 perception, by activating the transcriptional activator of multiple host defense genes NFkB, the production of interleukin (IL)-12 and the respiratory burst [35–39]. Lipopeptides are also involved in the stimulation of innate immunity in plants [40]. It is quite recent that RLs have been shown to be involved in triggering plant and animal defense responses and can be described as a new class of MAMPs.

### 3.1. Rhamnolipids as Stimulators of Human and Animal Immunity

RLs have been long known as exotoxins produced by the human pathogen *P. aeruginosa* [41–44] and several recent papers have highlighted their role in the stimulation of innate immunity in animal cells. The heat-stable Rha-Rha-C_14_-C_14_ produced by *Burkholderia plantarii* and some synthetic derivatives have been particularly studied [45–47]. Rha-Rha-C_14_-C_14_ is structurally quite similar to the RL exotoxin from *P. aeruginosa* and identical to the RL of *Burkholderia pseudomallei*, the causative agent of melioidosis, an infectious disease of humans and animals leading to skin infection, lung nodules and pneumonia [45]. This RL exhibits strong stimulatory activity on human mononuclear cells to produce TNFα, a pleiotropic inflammatory cytokine. Such a property has not been noted so far for RL exotoxins but only for the lipopolysaccharide (LPS) bacterial endotoxins. Like LPS, the cell stimulating activity of this RL could be inhibited by incubation with polymyxin B. Interestingly, immune cell activation by Rha-Rha-C_14_-C_14_ does not occur via receptors that are involved in LPS (TLR4) or lipopeptide signaling (TLR2) [45]. Synthetic RLs derived from *B. plantarii* Rha-Rha-C_14_-C_14_ were also analyzed for their immune cell activation [47]. These synthetic RLs differ by variations in the length, stereochemistry, number of lipid chains, number of rhamnoses and the occurrence of charged or neutral groups. The authors also compared these synthetic RLs to the well-characterized LPS MAMP from *Salmonella minnesota*. Immunostimulatory properties of RLs were monitored by assaying the secretion of TNFα and the induction of chemiluminescence in monocytes. Howe *et al*. [47] found that biological test systems showed large variations, depending on particular chemical structures and physicochemical parameters. LPS were, however, more efficient to induce luminescence and TNFα production than the RLs tested. Furthermore, they found that biologically inactive RLs with lamellar aggregate structures antagonize the induced activity in a way similar to lipid A-derived antagonists of LPS [47]. An extended study on structure-activity relationships of synthetic RLs derivatives also indicated a specific, recognition-based mode of action, with small structural variations in the RLs resulting in strong effects on the immunostimulatory activities [46]. RLs also stimulated the release of interleukin (IL)-8, granulocyte-macrophage colony-stimulating factor, and IL-6 from nasal epithelial cells at non-cytotoxic levels [48]. Interestingly, it was recently demonstrated that RLs could also potentiate the recognition of other MAMPs by the human innate immune system. Several MAMPs of *P. aeruginosa* are known to activate the innate immune system in epithelial cells, particularly the production of antimicrobial peptides such as the human beta-defensin-2 (hBD-2) and proinflammatory cytokines such as interleukin (IL)-8 [49]. In this study, RLs were found to interact with the well-known MAMP flagellin. The authors provide evidence that RLs are responsible for the release of flagellin from the flagella. Their findings indicate that upon adhesion to surfaces, *P. aeruginosa* may alter the outer membrane composition in an RL-dependent manner, thereby shedding flagellin from the flagella. In turn, epithelial cells recognize flagellin leading to synthesis of anti-microbial peptides as well as recruitment of inflammatory cells by induction of proinflammatory cytokines [49].

### 3.2. Rhamnolipids as Stimulators of Plant Immunity

RLs have very recently been characterized as new MAMPs involved in non-specific immunity in plants. They have been also shown to induce resistance in plants, which is effective against a broad range of pathogens [23]. It is demonstrated that Rha-C_10_-C_10_ and Rha-Rha-C_10_-C_10_ from *P. aeruginosa* and Rha-Rha-C_14_-C_14_ from *B. plantarii* trigger strong defense responses in grapevine including early events of cell signaling like Ca^2+^ influx, reactive oxygen species (ROS) production and MAP kinase activation. These RLs also induce a large battery of defense genes including some pathogenesis-related protein genes and genes involved in oxylipins and phytoalexins biosynthesis pathways [23]. Interestingly, depending on the concentrations tested, RLs were able to activate a programmed cell death reminiscent of animal apoptosis [23]. It was also demonstrated that RLs potentiate defense responses induced by other elicitors (*i.e.*, chitosan and a culture filtrate of the fungus *B. cinerea*). Another novel role of RLs consists in protecting grapevine against the necrotropic pathogen *B. cinerea*. RLs are also active in other plant species. They are able to stimulate defense genes in tobacco, wheat and *Arabidopsis thaliana* (Sanchez, L. unpublished work, 2010). RLs are also potent protectors in monocotyledonous plants against biotrophic fungi (Couleaud, G. Arvalis. Private communication, 2009). To date, it is not known whether the perception of RLs requires specific receptors in the plant plasma membrane [23]. Interestingly, lipopeptide biosurfactants, which are lipid derivatives with similar properties to RLs, have also been described as potent MAMP elicitors. Surfactin, the most studied cyclic lipopeptide from *Bacillus subtilis,* has been shown to trigger early signaling events and late defense responses in tobacco cell suspensions [50]. Some cyclic lipopeptides including Massetolide A and fengycin originating, respectively, from *Pseudomonas fluorescens* SS101 and *B. subtilis* S499 were identified as elicitors inducing a systemic resistance in tomato and bean [51,52]. As for RLs, it is yet unclear whether the induction of defense responses by lipopeptides requires specific receptors in the plant plasma membrane [40]. An alternative hypothesis is that lipopeptides could induce defense responses by membrane disturbance [50,53] and this could also be the case for RLs.

## 4. Potential Use of Rhamnolipids in Agricultural and Biomedical Fields

Major breakthroughs allowing production, separation and purification of RLs in industrial quantities and laboratory purities have allowed the application of these molecules in different fields from cosmetic to industrial and more recently from agriculture to medicine. As previously stated, the major advantage of using RL biosurfactants, which have diverse roles in plant and animal systems, is that they are natural and organic biodegradable compounds, originating from a large number of bacteria [1]. RLs have also been proposed to be used in food industry applications [12]. RLs have a direct biocide action on bacteria and fungi. They also increase the susceptibility of certain Gram-positive bacteria to specific antibiotics. RLs have been demonstrated to control zoosporic pathogens through lysis of their zoospores [21]. Clinical trials using RLs for the treatment of psoriasis, lichen planus, neurodermatitis and human burn wound healing have confirmed excellent ameliorative effects of RLs when compared to conventional therapy using corticosteroids [54,55]. RLs also display differential effects on human keratinocyte and fibroblast cultures [55]. The advantages of these biosurfactants are low irritancy and even anti-irritating effects, as well as compatibility with human skin [55]. Moreover, RLs have permeabilizing effects on Gram-positive and Gram-negative human bacterial strains, reinforcing their potential in biomedicine [20]. An important issue to be taken into account is the study of side effects of biosurfactants on plants and animals. Attention should be paid while using surfactants on plants as the latter could be affected in many different ways. Parameters like negative impact on crop yield or other important agronomical traits should not be neglected and should be studied in parallel to avoid any impact on plant growth or metabolism, while boosting plant immunity. For instance, it is known that high concentrations of RLs cause necrosis in plants [23]. Dose/response experiments in the field are a necessity in order to ensure use of non-toxic concentrations of RLs. In addition, in animal systems, RLs are known as virulence factors especially for immunocompromised patients and individuals suffering from cystic fibrosis (CF) [1]. At some concentrations, RLs also have hemolytic activity [56,57]. Thus, care should be taken in the use of RLs, albeit some applications such as fungicide and bactericide are already considered especially for skin treatments [54,55].

## 5. Conclusion

RLs are new actors in animal and plant defense and their low toxicity and biodegradability make them promising molecules to be used against pathogens. In this respect, there are some clues now available for the success of RL applications in greenhouses to fight phytopathogens. A better understanding of RL mode of action, especially their perception and the signaling pathways activated, will be very important to potentiate their beneficial effects in plants. RLs have a dual mode of action: they are antimicrobial and also stimulate plant defense responses. This dual property is probably very important for the efficiency of new biopesticides. In animals, the use of RLs is also at an advanced stage. RLs are successfully used as antimicrobial agents, especially for skin disease treatment. Deep insight into the physiochemical effects of RLs and their biological importance would reveal new dimensions in the fields of research like agriculture and medicine, precisely in plant defense, disease control and pathogenesis. An understanding of bacterial genera producing RLs that are not yet well studied would provide light on these fascinating aspects.

## Figures and Tables

**Table 1.** Antimicrobial properties of rhamnolipids.

**Organisms affected**	**Observed effects**	**RL application**	**RL origin**	**Ref.**
Fungi				
*Alternaria alternata*	growth inhibition (MIC)	RL mixture: Rha-Rha-C_10_-C_10,_ Rha-C_10_-C_10_, Rha-Rha-C_10_-C_12:1_	P. aeruginosa LBI	[9]
*Alternaria mali*	growth inhibition (MIC)	Rha-Rha-C_10_-C_10_	P. aeruginosa strain B5	[17]
*Aspergillus niger*	growth inhibition (MIC)	RL mixture: Rha-Rha-C_10_-C_10,_ Rha-C_10_-C_10_, Rha-Rha-C_10_-C_12:1_	P. aeruginosa LBI	[9]
*Aureobasidium pullulans*	growth inhibition (MIC)	RL mixture: Rha-Rha-C_10_-C_10,_ Rha-C_10_-C_10_, Rha-Rha-C_10_-C_12:1_	P. aeruginosa LBI	[9]
*Botrytis cinerea*	growth inhibition (MIC)	Rha-Rha-C_10_-C_10_	P. aeruginosa strain B5	[17]
	growth inhibition (MIC)	RL mixture: Rha-Rha-C_10_-C_10,_ Rha-Rha-C_10_-C_12_, Rha-C_10_-C_10_, Rha-Rha-C_10_-C_12:1_	P. aeruginosa 47T2	[10]
	inhibition of spore germination and mycelium growth	RL mixture: Rha-Rha-C_10_-C_10_, Rha-C_10_-C_10_ (Jeneil Biosurfactant Company JBR599)	*P. aeruginosa*	[23]
*Candida albicans*	growth inhibition (MIC)	RL mixture: Rha-Rha-C_10_-C_10,_ Rha-C_10_-C_10_, Rha-Rha-C_10_-C_12:1_	P. aeruginosa LBI	[9]
*Cercospora kikuchii*	growth inhibition (MIC)	Rha-Rha-C_10_-C_10_	P. aeruginosa strain B5	[17]
*Chaetonium globosum*	growth inhibition (MIC)	RL mixture: Rha-Rha-C_10_-C_10,_ Rha-Rha-C_10_-C_12_, Rha-C_10_-C_10_, Rha-Rha-C_10_-C_12:1_	P. aeruginosa 47T2	[10]
	growth inhibition (MIC)	RL mixture: Rha-Rha-C_10_-C_10,_ Rha-C_10_-C_10_, Rha-Rha-C_10_-C_12:1_	P. aeruginosa LBI	[9]
*Cladosporium cucumerinum*	growth inhibition (MIC)	Rha-Rha-C_10_-C_10_	P. aeruginosa strain B5	[17]
*Colletotrichum orbiculare*	growth inhibition (MIC)	Rha-Rha-C_10_-C_10_	P. aeruginosa strain B5	[17]
*Cylindrocarpon destructans*	growth inhibition (MIC)	Rha-Rha-C_10_-C_10_	P. aeruginosa strain B5	[17]
*Didymella bryoniae*	growth inhibition (MIC)	Rha-Rha-C_10_-C_10_	P. aeruginosa strain B5	[17]
*Fusarium solani*	growth inhibition (MIC)	RL mixture: Rha-Rha-C_10_-C_10,_ Rha-Rha-C_10_-C_12_, Rha-C_10_-C_10_, Rha-Rha-C_10_-C_12:1_	P. aeruginosa 47T2	[10]
*Fusarium sp.*	growth inhibition (MIC)	Rha-Rha-C_10_-C_10_	P. aeruginosa strain B5	[17]
	growth inhibition (MIC)	RL mixture: Rha-Rha-C_10_-C_10,_ Rha-Rha-C_10_-C_12_, Rha-C_10_-C_10_, Rha-Rha-C_10_-C_12:1_	P. aeruginosa 47T2	[10]
*Gliocadium virens*	growth inhibition (MIC)	RL mixture: Rha-Rha-C_10_-C_10,_ Rha-Rha-C_10_-C_12_, Rha-C_10_-C_10_, Rha-Rha-C_10_-C_12:1_	P. aeruginosa 47T2	[10]
	growth inhibition (MIC)	RL mixture: Rha-Rha-C_10_-C_10,_ Rha-C_10_-C_10_, Rha-Rha-C_10_-C_12:1_	P. aeruginosa LBI	[9]
*Magnaporthe grisea*	growth inhibition (MIC)	Rha-Rha-C_10_-C_10_	P. aeruginosa strain B5	[17]
*Mucor miehei*	growth inhibition (MIC)	RL mixture: Rha-Rha-C_10_-C_10,_ Rha-C_10_-C_10_	P. aeruginosa LBI	[12]
*Neurospora crassa*	growth inhibition (MIC)	RL mixture: Rha-Rha-C_10_-C_10,_ Rha-C_10_-C_10_	P. aeruginosa LBI	[12]
*Penicillium funiculosum*	growth inhibition (MIC)	RL mixture: Rha-Rha-C_10_-C_10,_ Rha-Rha-C_10_-C_12_, Rha-C_10_-C_10_, Rha-Rha-C_10_-C_12:1_	P. aeruginosa 47T2	[10]
	growth inhibition (MIC)	RL mixture: Rha-Rha-C_10_-C_10,_ Rha-C_10_-C_10_, Rha-Rha-C_10_-C_12:1_	P. aeruginosa LBI	[9]
*Phytophthora sp.*	zoospore lysis by RL intercalation into membrane	RL mixture: Rha-Rha-C_10_-C_10,_ Rha-C_10_-C_10_	*P. aeruginosa*	[21]
	growth inhibition (MIC), lytic effect on zoospores	Rha-Rha-C_10_-C_10_	P. aeruginosa strain B5	[17]
	zoospore motility inhibition, zoospore lysis, hyphae growth inhibition	nd	nd	[22]
	reduction of disease incidence and of disease severity	biosurfactant PRO1 (formulation of 25% Rls) Plant support (the Netherlands)	*P. aeruginosa*	[16]
	reduction of damping-off disease	RL mixture: Rha-Rha-C_10_-C_10_, Rha-Rha-C_10_-C_10:1_, Rha-C_10_-C_10_, Rha-Rha-C_10_-C_12:1_, Rha-C_10_-C_12:1_, Rha-C_10_-C_12_, Rha-Rha-C_10-_C_12_, Rha-Rha-C_10_-C_8_, Rha-C_8_-C_10_, Rha-Rha-C_8_-C_10_, Rha-Rha-C_12_-C_12_, Rha-Rha-C_12_-C_12:1_)	Pseudomonas sp. GRP3	[19]
*Pythium sp.*	zoospore lysis by RL intercalation into membrane	nd	*P. aeruginosa*	[21]
	zoospore motility inhibition, zoospore lysis, hyphae growth inhibition	nd	nd	[22]
	reduction of damping-off disease	RL mixture: Rha-Rha-C_10_-C_10_, Rha-Rha-C_10_-C_10:1_, Rha-C_10_-C_10_, Rha-Rha-C_10_-C_12:1_, Rha-C_10_-C_12:1_, Rha-C_10_-C_12_, Rha-Rha-C_10-_C_12_, Rha-Rha-C_10_-C_8_, Rha-C_8_-C_10_, Rha-Rha-C_8_-C_10_, Rha-Rha-C_12_-C_12_, Rha-Rha-C_12_-C_12:1_)	Pseudomonas sp. GRP3	[19]
	mycelial growth inhibition, reduction of disease symptoms, hyphae damages	RL-deficient mutant	P. aeruginosa PA01	[18]
*Rhizoctonia solani*	growth inhibition (MIC)	Rha-Rha-C_10_-C_10_	P. aeruginosa strain B5	[17]
	growth inhibition (MIC)	RL mixture: Rha-Rha-C_10_-C_10,_ Rha-Rha-C_10_-C_12_, Rha-C_10_-C_10_, Rha-Rha-C_10_-C_12:1_	P. aeruginosa 47T2	[10]
Bacteria				
Gram-negative				
*Enterobacter aerogenes*	growth inhibition (MIC)	RL mixture: Rha-Rha-C_10_-C_10,_ Rha-Rha-C_10_-C_12_, Rha-C_10_-C_10_, Rha-Rha-C_10_-C_12:1_	P. aeruginosa 47T2	[10]
	growth inhibition (MIC)	RL mixture: Rha-Rha-C_10_-C_10,_ Rha-C_10_-C_10_, Rha-Rha-C_10_-C_12:1_	P. aeruginosa LBI	[9]
*Erwinina carotovora*	growth inhibition (MIC)	RL mixture: Rha-Rha-C_10_-C_10,_ Rha-Rha-C_10_-C_12_, Rha-C_10_-C_10_, Rha-Rha-C_10_-C_12:1_	P. aeruginosa 47T2	[10]
*Escherichia coli*	growth inhibition (MIC)	RL mixture: Rha-Rha-C_10_-C_10,_ Rha-Rha-C_10_-C_12_, Rha-C_10_-C_10_, Rha-Rha-C_10_-C_12:1_	P. aeruginosa 47T2	[10]
	growth inhibition (MIC)	nd	P. fluorescens HW-6	[13]
*Klebsiella pneumoniae*	growth inhibition (MIC)	RL mixture: Rha-Rha-C_10_-C_10,_ Rha-Rha-C_10_-C_12_, Rha-C_10_-C_10_, Rha-Rha-C_10_-C_12:1_	P. aeruginosa 47T2	[10]
*Proteus mirabilis*	growth inhibition (MIC)	RL mixture: Rha-Rha-C_10_-C_10,_ Rha-C_10_-C_10_, Rha-Rha-C_10_-C_12:1_	P. aeruginosa LBI	[9]
*Pseudomonas aeruginosa*	growth inhibition (MIC)	RL mixture: Rha-Rha-C_10_-C_10,_ Rha-C_10_-C_10_, Rha-Rha-C_10_-C_12:1_	P. aeruginosa LBI	[9]
	increase in released proteins	Biosurfactant PS (rhamnolipid+alginate)	Pseudomonas sp. S-17	[20]
	reduction of LPS contents, increase in cell hydrophobicity and in extracellular protein release, changes in outer membrane proteins	Biosurfactant PS (rhamnolipid+alginate)	Pseudomonas sp. S-17	[15]
	growth inhibition, increase in cell permeability and in released proteins	nd	P. fluorescens HW-6	[13]
*Ralstonia solanacearum*	growth inhibition (MIC)	Rha-Rha-C_10_-C_10_	P. aeruginosa strain B5	[17]
*Salmonella thyphimurium*	growth inhibition (MIC)	RL mixture: Rha-Rha-C_10_-C_10,_ Rha-C_10_-C_10_, Rha-Rha-C_10_-C_12:1_	P. aeruginosa LBI	[9]
*Serratia marcescens*	growth inhibition (MIC)	Rha-Rha-C_10_-C_10_	P. aeruginosa strain B5	[17]
	growth inhibition (MIC)	RL mixture: Rha-Rha-C_10_-C_10,_ Rha-Rha-C_10_-C_12_, Rha-C_10_-C_10_, Rha-Rha-C_10_-C_12:1_	P. aeruginosa 47T2	[10]
*Xanthomonas campestris*	growth inhibition (MIC)	Rha-Rha-C_10_-C_10_	P. aeruginosa strain B5	[17]
Gram-positive				
*Bacillus cereus*	growth inhibition (MIC)	RL mixture: Rha-Rha-C_10_-C_10,_ Rha-Rha-C_10_-C_12_, Rha-C_10_-C_10_, Rha-Rha-C_10_-C_12:1_	P. aeruginosa 47T2	[10]
	growth inhibition (MIC)	RL mixture: Rha-Rha-C_10_-C_10,_ Rha-C_10_-C_10_, Rha-Rha-C_10_-C_12:1_	P. aeruginosa LBI	[9]
	growth inhibition (MIC)	RL mixture: Rha-Rha-C_10_-C_10,_ Rha-C_10_-C_10_	P. aeruginosa LBI	[12]
*Bacillus sp.*	growth inhibition (MIC)	nd	P. fluorescens HW-6	[13]
*Bacillus subtilis*	growth inhibition (MIC)	RL mixture: Rha-Rha-C_10_-C_10,_ Rha-Rha-C_10_-C_12_, Rha-C_10_-C_10_, Rha-Rha-C_10_-C_12:1_	P. aeruginosa 47T2	[10]
	growth inhibition (MIC)	RL mixture: Rha-Rha-C_10_-C_10,_ Rha-C_10_-C_10_, Rha-Rha-C_10_-C_12:1_	P. aeruginosa LBI	[9]
*Micrococcus luteus*	growth inhibition (MIC)	RL mixture: Rha-Rha-C_10_-C_10,_ Rha-Rha-C_10_-C_12_, Rha-C_10_-C_10_, Rha-Rha-C_10_-C_12:1_	P. aeruginosa 47T2	[10]
	growth inhibition (MIC)	RL mixture: Rha-Rha-C_10_-C_10,_ Rha-C_10_-C_10_, Rha-Rha-C_10_-C_12:1_	P. aeruginosa LBI	[9]
	growth inhibition (MIC)	RL mixture: Rha-Rha-C_10_-C_10,_ Rha-C_10_-C_10_	P. aeruginosa LBI	[12]
*Staphylococcus aureus*	growth inhibition (MIC)	RL mixture: Rha-Rha-C_10_-C_10,_ Rha-Rha-C_10_-C_12_, Rha-C_10_-C_10_, Rha-Rha-C_10_-C_12:1_	P. aeruginosa 47T2	[10]
	growth inhibition (MIC)	RL mixture: Rha-Rha-C_10_-C_10,_ Rha-C_10_-C_10_	P. aeruginosa LBI	[12]
*Staphylococcus epidermidis*	growth inhibition (MIC)	RL mixture: Rha-Rha-C_10_-C_10,_ Rha-Rha-C_10_-C_12_, Rha-C_10_-C_10_, Rha-Rha-C_10_-C_12:1_	P. aeruginosa 47T2	[10]
*Streptococcus faecalis*	growth inhibition (MIC)	RL mixture: Rha-Rha-C_10_-C_10,_ Rha-C_10_-C_10_, Rha-Rha-C_10_-C_12:1_	P. aeruginosa LBI	[9]
Amoeba*(Dictyostelium discoideum)*	growth inhibition, cell lysis	Rhl quorum-sensing mutants	P. aeruginosa PA01	[24]
Algae*(Heterosigma akashiwo)*	growth inhibition, cell lysis, plasma membrane and organelles damages, condensation of chromatin	RL mixture: Rha-Rha-C_10_-C_10,_ Rha-C_10_-C_10_	*P. aeruginosa*	[29]
Virus				
potato virus X, red clover mottle virus	reduction of local lesions, reduction of virus number	nd	nd	[25]
herpes simplex virus HSV)	inhibition of cytopathic effects	biosurfactant PS-17 (rhamnolipid+alginate)	Pseudomonas sp. S-17	[27]

MIC: minimum inhibitory concentrations ; nd : not done or not communicated
